# Lateral Flow-Based Skin Patch for Rapid Detection of Protein Biomarkers in Human Dermal Interstitial Fluid

**DOI:** 10.1021/acssensors.4c00956

**Published:** 2024-10-25

**Authors:** Elizabeth C. Wilkirson, Danika Li, Peter B. Lillehoj

**Affiliations:** † Department of Mechanical Engineering, Rice University, Houston, Texas 77005, United States; ‡ Department of Bioengineering, Rice University, Houston, Texas 77030, United States

**Keywords:** lateral flow immunoassay, microfluidic, interstitial fluid, skin, diagnostic, tetanus, SARS-CoV-2

## Abstract

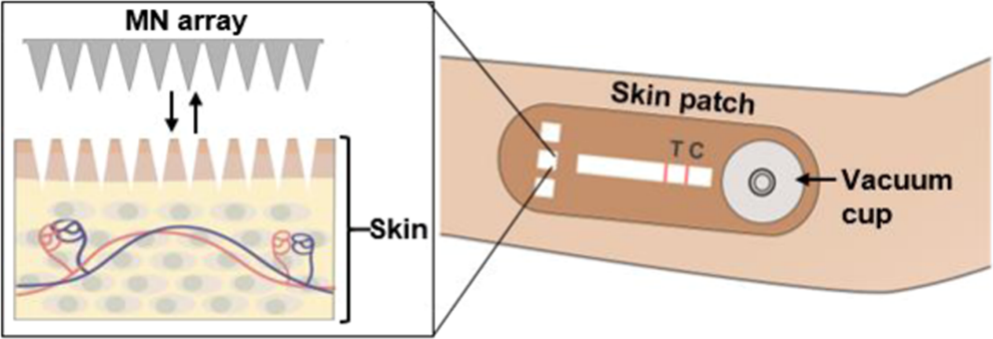

Rapid diagnostic tests (RDTs) offer valuable diagnostic information in a quick, easy-to-use and low-cost format. While RDTs are one of the most commonly used tools for in vitro diagnostic testing, they require the collection of a blood sample, which is painful, poses risks of infection and can lead to complications. We introduce a blood-free point-of-care diagnostic test for the rapid detection of protein biomarkers in dermal interstitial fluid (ISF). This device consists of a lateral flow immunochromatographic assay (LFIA) integrated within a microfluidic skin patch. ISF is collected from the skin using a microneedle array and vacuum-assisted extraction system integrated in the patch, and transported through the lateral flow strip via surface tension. Using this skin patch platform, we demonstrate in situ detection of anti-tetanus toxoid IgG and SARS-CoV-2 neutralizing antibodies, which could be accurately detected in human ISF in <20 min. We envision that this device can be readily modified to detect other protein biomarkers in dermal ISF, making it a promising tool for rapid diagnostic testing.

Rapid diagnostic testing plays an important role in medicine and is used for various applications, including the detection of current or past infections, monitoring disease progression or therapeutic response, and determining immune status to guide vaccination decisions. One of the most common types of rapid diagnostic tests (RDTs) are lateral flow immunochromatographic assays (LFIAs), which use antibody-conjugated gold nanoparticles (AuNPs) and a lateral flow technique to detect the presence of specific protein biomarkers in the blood of an individual with a visible line on a test strip. Due to their low-cost, ease of use, and quick turnaround times, RDTs are the most widely used tool in the world for the detection and diagnosis of infectious diseases, such as malaria, HIV infection and hepatitis. While RDTs have revolutionized medical diagnostic testing, they traditionally require the collection of a blood sample, which poses risks of infection^1^ and can lead to complications, particularly in newborns and individuals with blood clotting disorders.^2^ Additionally, the pain associated with blood sampling can deter individuals with blood or needle phobias from getting tested.^3,4^ While RDTs based on urine and saliva have been developed, these fluids offer limited diagnostic utility since they contain only subsets of the biomarkers found in blood^5^ and typically at significantly lower concentrations.^6^

Recently, there has been growing interest in the use of interstitial fluid (ISF), the fluid in the extracellular space in tissues, for diagnostic testing.^7^ ISF is abundant in the skin, which is comprised of up to 70% of ISF by volume,^8^ making it readily accessible for sampling. Microneedle- (MN)-based techniques have been demonstrated for minimally invasive sampling of ISF from skin (i.e., dermal ISF), which are safe and nearly pain-free.^9^ Dermal ISF has shown to have nearly the same analytes, including metabolites, proteins and RNA, as blood.^10−15^ For example, >90% of circulating proteins in blood are also present in dermal ISF^8,14^ suggesting that a significant number of blood-based biomarkers are also detectable in ISF. In addition to circulating proteins which are associated with systemic physiology, dermal ISF contains localized biomarkers associated with skin and tissue physiology that are not found in blood,^8,14,16^ making it potentially useful for the diagnosis of skin diseases, conditions and disorders.

Owing to the rich biomolecular content of ISF, MN-based platforms have been developed to collect human ISF for ex situ analysis. Hydrogel-forming MNs made from cross-linked poly(methyl vinyl ether-*co*-maleic acid) were used by Caffarel-Salvador et al. to extract dermal ISF from human skin.^17^ In this approach, MNs were applied to the skin for up to 3 h to allow the hydrogel to swell with ISF. After being removed from the skin, the ISF was extracted from the MNs and analyzed for glucose and caffeine using isocratic high-performance liquid chromatography. Ribet et al. developed an ISF sampling device consisting of a hollow MN and microfluidic chip.^11^ In this approach, the ISF sample was stored in an analytical-grade paper matrix in a dry format, enabling it to be mailed to a centralized laboratory for biomolecular analysis. The dried ISF sample was extracted from the paper and analyzed for caffeine, SARS-CoV-2 antibodies and other proteins using liquid chromatography-tandem mass spectrometry, a proximity extension assay, and a single-molecule array assay. Samant et al. demonstrated the extraction of ISF from human skin using solid metal MNs and an electric vacuum pump.^8,15^ Using this method, ∼170 clinically relevant metabolites were identified in dermal ISF obtained from volunteers via liquid chromatography mass spectrometry (LCMS). In another approach, a sampling device consisting of hollow MNs connected to capillary tubes was applied to the forearm of volunteers for 1–2 h for ISF extraction. Proteomic analysis using LCMS revealed the presence of >3000 proteins in the extracted ISF samples.^10,14^ While these studies demonstrate the detection of clinically relevant analytes in human ISF, they involve time-consuming (>1 h) sampling procedures or tedious protocols to extract/elute ISF from MNs, and require sophisticated instrumentation for biomolecular analysis, all of which hinders their utility for point-of-care testing.

MN-based biosensing platforms have also been developed for transdermal detection and monitoring of analytes in human ISF. Zhu et al. reported a polymeric swellable MN patch coupled with an electrode array for potentiometric measurements of ions (Na^+^, K^+^, Ca^2+^) in human ISF.^18^ A wearable MN- and field-effect transistor-based biosensor was developed by Zheng et al. and used for measurements of Na^+^ in human ISF.^19^ Friedel et al. developed an aptamer-based electrochemical sensor for continuous measurements of phenylalanine in human ISF.^20^ Continuous monitoring of metabolites (e.g., glucose, lactate) in dermal ISF has also been demonstrated using MN-based electrochemical sensors.^21−24^ An integrated wearable device consisting of a MN array, electrochemical sensors, a miniature potentiostat and battery was reported by Tehrani et al. for continuous measurements of lactate, glucose, and alcohol in human ISF.^25^ While these platforms demonstrate the ability to perform in situ measurements of analytes in dermal ISF, they required the use of bulky and/or specialized electronic components, such as electrochemical analyzers or custom circuits, and were limited to the detection of small molecules (e.g., metabolites, drugs).

Here, we report a point-of-care diagnostic test for rapid in situ detection of protein biomarkers in dermal ISF, which offers an instrument-free colorimetric readout that can be interpreted by the naked eye. In this approach, an MN array (Figure S1A,B) is used to generate micropores in skin from which ISF is extracted and transported into the skin patch using an integrated vacuum-assisted extraction system. For the first proof of principle demonstration, this device was designed to detect anti-tetanus toxoid IgG, which has clinical relevance in determining an individual’s immunity to tetanus infection. Measurements of anti-tetanus toxoid IgG in dermal ISF and blood obtained from four volunteers were performed using a commercial enzyme-linked immunosorbent assay (ELISA) kit to determine the anti-tetanus toxoid IgG levels in both fluids. The functionality of this device was evaluated by testing it on a volunteer, which revealed its ability to accurately detect anti-tetanus toxoid IgG in dermal ISF in <20 min. We also modified the skin patch for the detection of SARS-CoV-2 neutralizing antibodies and tested its functionality on another volunteer, showcasing its broad applicability for protein biomarker detection in dermal ISF. In addition to its quick turnaround time and ease of use, the only equipment required is a vacuum cup and hand pump (which are inexpensive [<$10] and widely available), making this device well suited for point-of-care diagnostic testing, particularly in resource-limited settings.

## Experimental Section

### Design and Fabrication of the MN Array

The MN array is comprised of solid, conical MNs made from polymerized SU-8 photoresist coated with 1.5 μm of parylene for enhanced mechanical strength and biocompatibility.^26,27^ The MNs were designed to have a compact profile to minimize the discomfort when inserted into skin. Each MN has a base diameter of 200 μm and height of 450 μm, which was determined in a prior study to be the optimal MN dimensions for collecting the greatest amount of ISF from human skin via vacuum-assisted extraction.^28^ The MNs are configured in a two-dimensional 10 × 10 array to multiply the number of micropores generated per insertion, with a needle-to-needle spacing of 400 μm. The overall size of the MN array is 7.5 × 7.5 mm.

The MN array master was printed on a Photonic Professional GT lithography system (NanoScribe, MA, USA). MN array replicas were fabricated via centrifugation-assisted replica molding where master molds were constructed from polydimethylsiloxane (PDMS) (Sylgard 184, Dow, MI, USA) mixed at a 1:10 (curing agent-to-elastomer) ratio. The PDMS was degassed for 30 min, poured over the master, and heated in a convection oven at 80 °C for 2 h. Cured PDMS was cut into individual molds using a razor blade and cleaned in 70% isopropanol. SU-8 2025 photoresist (Kayaku Advanced Materials, MA, USA) was poured into the PDMS molds and centrifuged at 4000*g* for 15 min to create MN array replicas. Replicas were cured under a 50W UV (365 nm) lamp for 3 min, then coated with 1.5 μm of parylene using a Labcoater 2 parylene deposition system (Specialty Coating Systems, IN, USA). Three mm-thick poly(methyl methacrylate) (PMMA) (McMaster Carr, IL, USA) was attached to the backside of the MN arrays to enhance their rigidity. MNs were inspected and imaged using a VHX-7000 optical microscope (Keyence Corporation, Osaka, Japan).

### Preparation of AuNP–Anti-human IgG Conjugates

200 μL of 30 nm-diameter AuNPs in solution (OD-50; Millipore Sigma, MA, USA) was aliquoted into a low-bind microcentrifuge tube (Eppendorf, Hamburg, Germany). 1-Ethyl-3-(3-(dimethylamino)propyl)carbodiimide (EDC) (Thermo Fisher Scientific, MA, USA) and *N*-hydroxysuccinimide (NHS) (Thermo Fisher Scientific) were prepared at 10 mg/mL in deionized water. 40 μL of EDC and 80 μL of NHS were added to the AuNP solution, incubated on a shaker at room temperature for 30 min, and then centrifuged at 10,000*g* for 10 min. The supernatant was removed, and the pellet was resuspended in 200 μL of reaction buffer to wash away excess EDC and NHS. The solution was vortexed and centrifuged at 10,000*g* for 10 min. The supernatant was removed and 200 μL of fresh reaction buffer was added. 1.55 μL of goat anti-human IgG (1.3 mg/mL; Jackson ImmunoResearch, PA, USA) was added to the solution, followed by 3 h of incubation on a shaker at room temperature. After incubation, 2 μL of quencher was added and incubated for 10 min, followed by 10 min of centrifugation at 10,000*g*. The supernatant was removed, and the pellet was resuspended in 200 μL of reaction buffer. The AuNP–anti-human IgG conjugate concentration was adjusted to OD-20 by adding 500 μL of conjugate diluent to the AuNP solution. Prepared AuNP–anti-human IgG conjugate solution was stored at 4 °C.

### Preparation of the Conjugate Release Pad

Glass fiber strips (Millipore Sigma, MA, USA) were soaked in a phosphate-buffered saline (PBS) solution containing 10% sucrose (Millipore Sigma) in PBS, 2% bovine serum albumin (BSA) (Millipore Sigma) in PBS, and 0.25% Tween-20 (Millipore Sigma) in deionized water for 1 h at 4 °C. The strips were dried at 37 °C for 2 h and hand-cut into 3 mm-wide pads. Three μL of AuNP–anti-human IgG conjugate solution was dispensed onto the conjugate release pads, dried at 37 °C for 2 h and stored at 4 °C with desiccant.

### Preparation of the Nitrocellulose Membrane

Nitrocellulose membrane (GE Healthcare, IL, USA) was adhered to a 60 mm × 300 mm backing card (DCN Dx, CA, USA). For anti-tetanus toxoid IgG detection, solutions of tetanus toxoid antigen reconstituted in deionized water (3 mg/mL; Enzo Life Sciences, NY, USA) and rabbit anti-goat IgG (H/L) in PBS (1 mg/mL; Bio-Rad Antibodies, CA, USA) were dispensed onto the membrane to generate test and control lines, respectively, using an automated liquid dispensing platform (BioDot XYZ3060, CA, USA). For SARS-CoV-2 neutralizing antibody detection, solutions of SARS-CoV-2 spike glycoprotein S1 reconstituted in deionized water (0.5 mg/mL, Abcam, Cambridge, U.K.) and rabbit anti-goat IgG (H/L) in PBS (1 mg/mL; Bio-Rad Antibodies) were drawn onto the membrane using a fine-tip paint brush (Zem Brush, Ohio, USA) for the test and control lines, respectively. The membranes were dried at 37 °C for 2 h.

### Assembly of the Lateral Flow Test Strip

A 3 mm × 20 mm cellulose absorbent pad (Millipore Sigma) was adhered to the backing card slightly overlapping (∼1 mm) the end of the prepared nitrocellulose membrane. The card was cut into 3 mm-wide strips using a guillotine cutter (BioDot, CA, USA). Prepared strips were stored at 4 °C with desiccant.

### Fabrication and Assembly of the Skin Patch

The skin patch was designed using AutoCAD (Autodesk, CA, USA) and Solidworks (SolidWorks Corp., MA, USA) software. The microfluidic substrate was fabricated from 3 mm-thick PMMA (McMaster Carr) and microchannels were etched into the substrate using a CNC micromilling machine (Minitech Machinery Corporation, GA, USA). A CO_2_ laser cutter (Universal Laser System, Inc., AZ, USA) was used to create the vacuum and sampling ports in the microfluidic substrate, double-sided pressure-sensitive tape (3M, MN, USA), poly(ethylene terephthalate) (PET) film (Optiazure) and bandage tape (3M). The LFIA test strip was inserted into the microfluidic substrate and a prepared conjugate release pad was placed at the front of the strip. The assembled test strip was enclosed within the patch using PET film and double-sided pressure-sensitive tape. The PMMA-LFIA-PET assembly was sandwiched between two layers of medical-grade tape, securing it within the patch.

### MN Penetration Testing

Cadaver porcine skin with hair, fat and subcutaneous tissue removed was purchased from Animal Technologies, Inc. (TX, USA). The skin was cut into 10 cm × 10 cm sections, vacuum sealed, and stored at −20 °C. Prior to testing, a frozen skin section was thawed at room temperature and mounted onto foil-wrapped cardboard using safety pins. MNs were coated in blue ink and the MN array was inserted into the skin section using a MN applicator (Micropoint Technologies, Singapore). MN insertion wounds were visualized using a Keyence VHX-7000 microscope.

Histological analysis was performed on porcine skin sections following MN insertion. MNs were coated with Trypan blue (Sigma-Aldrich, MA, USA) in glycerol (Sigma-Aldrich) solution, and the MN array was inserted into the skin section using a MN applicator. The skin sample was fixed in a 10% formalin solution (Sigma-Aldrich) for at least 48 h, transferred and stored in a 70% ethanol solution. The sample was then embedded in paraffin (Sigma-Aldrich), dehydrated, sectioned, and stained with hematoxylin and eosin (H&E). Optical images of H&E-stained skin sections were captured using a Keyence VHX-7000 microscope.

### ISF and Blood Collection from Human Volunteers

Dermal ISF and fingerstick blood was collected from volunteers, which was performed under the guidance and approval from the Rice University Institutional Review Board (IRB-FY2021-147). Potential participants were provided with informed consent to participate in the study. Participants were explained the entirety of the sample collection process prior to beginning the study and informed consent was obtained from each individual. Criteria for participation was as follows: healthy adults or Rice University students ages 18 or older with no blood clotting disorders (including hemophilia, or factor II, V, VII, X, or XII deficiencies) or known skin allergies to medical adhesives. For ISF collection, the participant’s forearm was cleaned using an alcohol prep pad (Fisher Healthcare, MA, USA). An adhesive stencil with cutouts for the MN insertion sites was adhered to the forearm and the MN array was applied two times at the insertion sites using a MN applicator (Figure S2A). A rigid PMMA plate with cutouts at the MN insertion sites was attached to the stencil, followed by the attachment of a vacuum cup (Figure S2B). Vacuum pressure was generated inside the cup using a hand pump (Hansol Medical, South Korea) (Figure S2C). After 20 min, the vacuum cup was removed and the extracted ISF was collected using capillary tubes (Thermo Fisher Scientific, MA, USA and Drummond Scientific Company, PA, USA) (Figure S2D). The collected ISF was transferred to a low-bind microcentrifuge tube, incubated at room temperature for 1 h and centrifuged at 10,000*g* for 10 min. The supernatant was collected for analysis. Blood samples were obtained via fingerstick using a lancing device (Bayer Microlet) and 30G lancets (CareTouch). Blood was collected in capillary tubes, transferred to a low-bind microcentrifuge tube, incubated for 1 h at room temperature, and centrifuged at 10,000*g* for 10 min. The purified serum sample was transferred to a new low-bind microcentrifuge tube for analysis.

### Anti-tetanus Toxoid IgG Quantification in Blood and ISF Samples

Anti-tetanus toxoid IgG levels were measured in dermal ISF and blood samples using a human anti-tetanus toxoid IgG ELISA kit (Alpha Diagnostics, TX, USA). Measurements were performed according to the manufacturer’s instructions. The absorbance values were measured at 450 nm using a Biotek Epoch absorbance reader. A standard curve was calculated and used to determine the anti-tetanus toxoid IgG concentration in the samples.

### Fluid Flow Characterization through the Skin Patch

We assessed the fluid flow characteristics through the skin patch using an artificial skin model.^29^ Briefly, 2% agar gel (Sigma-Aldrich) solution was boiled, poured into a 100 mm Petri dish, cured at room temperature, and stored at 4 °C. Blue dye solution was dispensed on top of the agar gel and covered by Parafilm (Bemis Company, Inc., WI), which was carefully stretched over the Petri dish to prevent the entrapment of air bubbles. To initiate the experiment, a MN array was applied to the artificial skin using a MN applicator, followed by the attachment of the patch. A vacuum cup was attached to the vacuum port and vacuum pressure was generated using a hand pump. Video recordings and frame extractions were performed using an iPhone 14 Pro.

### Evaluating the Sensitivity and Selectivity of the Tetanus Lateral Flow Immunoassay

For sensitivity testing, 15 μL of tetanus toxoid standard IgG (Alpha Diagnostics) spiked in ISF simulant (10.3 mg/mL human serum albumin diluted in Tyrode’s salts^30^) at varying concentrations was dispensed onto the conjugate pad of the lateral flow test strip. For selectivity testing, measurements were performed by dispensing 15 μL of tetanus toxoid standard IgG (Alpha Diagnostics), diphtheria toxoid IgG (Virion, Wurzburg, Germany) or *Bordetella pertussis* toxin IgG (Virion) at 0.1 IU/mL onto the conjugate pad. Images of the test results were obtained using a Canon CanoScan 9000F scanner.

### In Situ Protein Detection in Dermal ISF Using the Skin Patch

Positive control measurements were carried out by testing the patch on volunteers with up-to-date vaccinations. The volunteers’ anterior forearm was first cleaned using an alcohol prep pad and the MN array was applied to the skin using a MN applicator. Next, the patch was adhered to the skin and a vacuum cup was attached to the vacuum port of the patch. Vacuum pressure was generated using a hand pump. After 18 min, a photograph of the test result was captured using an iPhone 14 Pro. Afterward, the vacuum cup was detached from the patch, the patch was peeled off and the forearm was cleaned using a new alcohol prep pad. Photographs of the volunteer’s forearm were captured at various time intervals after testing.

Negative control measurements were performed by testing the patch on cadaver porcine skin (Animal Technologies, Inc.) with hair, fat and subcutaneous tissue removed. The skin was dermally injected with 20 μL of ISF simulant using a syringe. The MN array was applied to the skin, followed by the attachment of the skin patch and vacuum cup, and application of vacuum pressure. After 18 min, a photograph of the test result was captured using an iPhone 14 Pro.

### Characterization of ISF Extraction Volume

We assessed the volume of dermal ISF extracted by the skin patch by testing it on volunteers. The volunteers’ anterior forearm was first cleaned using an alcohol prep pad and the MN array was applied to the skin using a MN applicator. Next, the patch was adhered to the skin and a vacuum cup was attached to the vacuum port of the patch. Vacuum pressure was generated using a hand pump. After 18 min, the vacuum cup was detached from the patch and the patch was removed from the skin. The mass of the LFIA test strip saturated with ISF was measured and compared to its dry mass (measured prior to the experiment), and the difference in mass was used to determine the volume based on the fluid density. The density of each ISF sample was calculated independently using a known mass and volume of fluid, which was collected using the method described above in “ISF and Blood Collection from Human Volunteers.”

## Results

### Design of the Skin Patch

**Figure 1:**
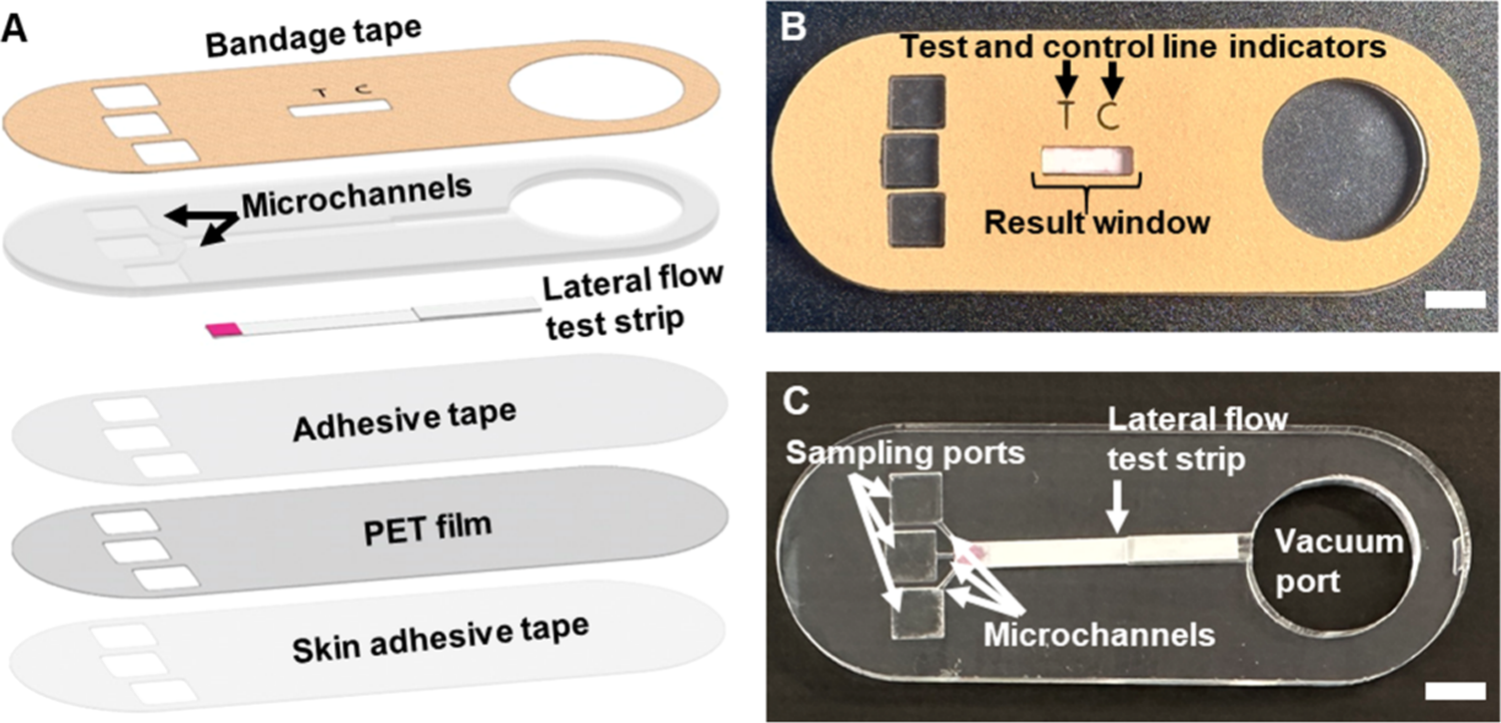
Overview of the skin patch design. (A) Exploded view depicting the individual layers of the patch. Topside (B) and internal (C) views of the assembled patch. Scale bars, 6 mm.

This device consists of a colloid gold-based LFIA integrated within a skin patch, which is comprised of a PMMA microfluidic network and PET film sandwiched between three layers of adhesive tape (Figure 1A). The lateral flow test strip is based on a conventional LFIA architecture,^31^ and consists of a glass fiber conjugate release pad, cellulose absorbent pad, and nitrocellulose membrane on a poly(vinyl chloride) backing card. The conjugate release pad contains AuNPs conjugated with anti-human IgG and the nitrocellulose membrane contains immobilized tetanus toxoid antigen and rabbit anti-goat IgG representing the test line and control line, respectively. During testing, anti-tetanus toxoid IgG in the sample binds to the AuNP–anti-human IgG conjugates on the conjugate pad forming AuNP–anti-human IgG–anti-tetanus toxoid IgG conjugates, which migrate toward the test line where they are captured to generate a red line. Uncaptured AuNP–anti-human IgG conjugates subsequently bind to the control line to generate a second red line, providing validation of a functioning test. The intensity of the test line is correlated with the concentration of anti-tetanus toxoid IgG in the sample where higher anti-tetanus toxoid IgG levels result in the generation of darker lines. Based on this detection scheme, this assay was designed to generate a test line (denoting a “positive” result) only when the anti-tetanus toxoid IgG concentration in the sample is equal or higher than the protective threshold concentration, indicating that the individual possesses sufficient protection against tetanus infection. When the anti-tetanus toxoid IgG concentration is below the protective threshold concentration, then no test line is generated (denoting a “negative” result), which indicates that the individual possesses inadequate immunity against tetanus infection and requires a booster vaccine.

The microfluidic network is fabricated from 3 mm-thick PMMA and contains cutouts for the fluidic channels, which are connected to three 6 mm × 6 mm sampling ports and an 18 mm-diameter vacuum port. The LFIA test strip is secured within a 3 mm-wide channel in the microfluidic network using the PET film and double-sided pressure-sensitive tape, and the PMMA-LFIA-PET assembly is sandwiched between two layers of medical-grade tape (the top layer is bandage tape, and the bottom layer is double-sided adhesive tape). The topside of the patch contains cutouts for the sampling ports (to facilitate alignment with the MN insertion sites), test (“T”) and control (“C”) line indicators, a test result window, and the vacuum port (Figure 1B). The internal view of the patch reveals the configuration of the sampling ports, microchannels, LFIA strip, and the vacuum port (Figure 1C). The adhesive backing enables the patch to remain securely attached to the skin during testing.

### Characterization of MN Penetration

The mechanical strength of the MN array was evaluated in a prior study, which revealed its ability to withstand up to 50 N of compression without exhibiting signs of failure (i.e., fracture) and be repeatedly inserted into porcine skin for at least 36 times with no discernible deformation or damage.^28^ We briefly assessed the capability of the MN array to generate micropores in skin via insertion into cadaver porcine skin, which was used as an anatomically and biochemically similar model to human skin.^32^ Prior to skin insertion, MNs were coated with blue ink for improved visualization. Distinct pores were generated by each MN, which were confined to the needle penetration sites with no impact to the surrounding tissue (Figure S1C). Histological analysis was performed to evaluate the effects of microneedle penetration in skin tissue. Each MN insertion site was characterized by a conical micropore that pierced through the epidermis (Figure S1D). The formation of these cavities provides access to ISF in the upper dermis, while avoiding the dense collection of nerves and vascular structures located in the lower dermis.^33^

### Analysis of Dermal ISF and Blood for Anti-tetanus Toxoid IgG

**Figure 2:**
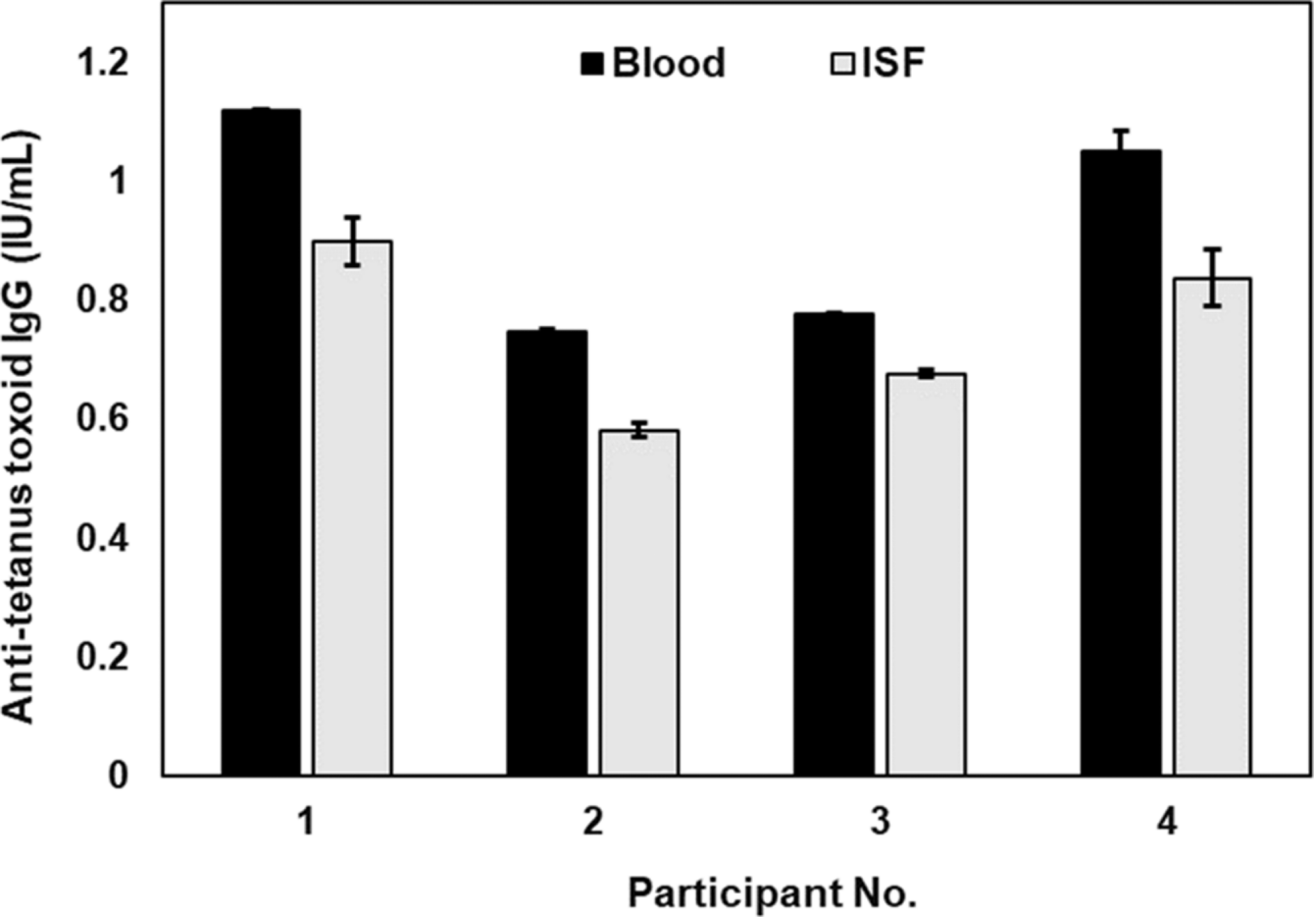
Anti-tetanus toxoid IgG levels in human blood and ISF. Concentration of anti-tetanus toxoid IgG in blood and dermal ISF sampled from four volunteers measured using a commercial ELISA kit. Each bar represents the mean ± standard deviation (SD) of two measurements (*n* = 2).

Paired dermal ISF and blood samples from four healthy volunteers (demographics are listed in Table S1) were analyzed for anti-tetanus toxoid IgG levels using a commercial ELISA kit. Antibodies to tetanus toxoid were detected in ISF of all the volunteers at concentrations from ∼0.6 to 1.1 IU/mL (Figure 2). Anti-tetanus toxoid IgG levels in ISF were well-correlated with those in blood where the average ISF-to-blood ratio for all the samples was ∼0.8 (i.e., ∼20% lower in ISF than blood). These results are consistent with prior experimental studies which show that levels of moderately large (∼100–150 kDa) antibodies in ISF are 15–39% lower than in blood.^34−39^ These collective results provide compelling evidence that immune antibodies generated in response to infections and vaccinations are present in dermal ISF.

### Fluid Flow through the Skin Patch

**Figure 3:**
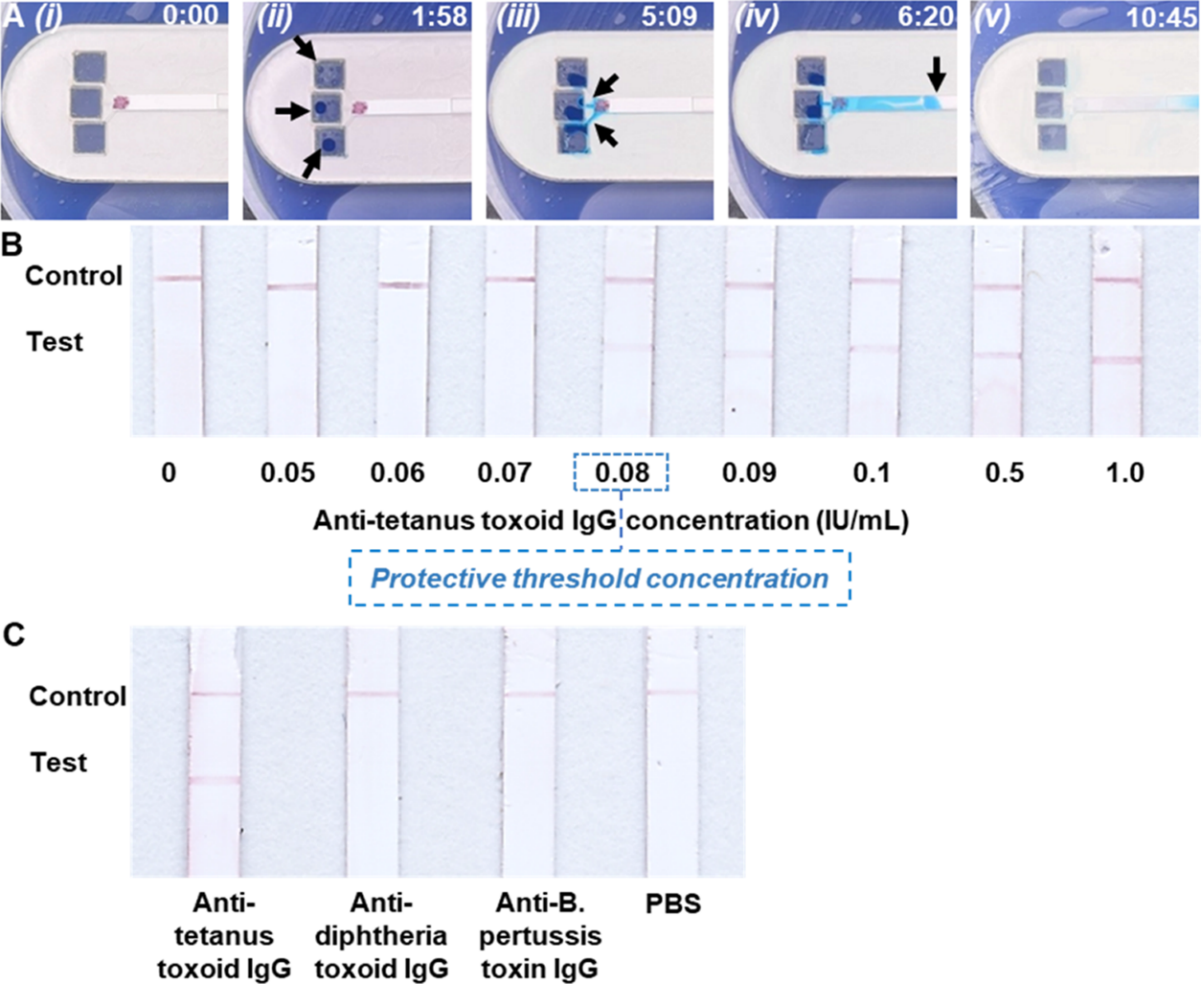
Characterization of the skin patch. (A) Sequential still frame images showing the extraction and transport of liquid through the patch (without the bandage tape) in an artificial skin model. Arrows indicate the location(s) of the liquid front. Time stamps (min:s) are in the upper right corner. (B) Test results of ISF samples with increasing concentration of anti-tetanus toxoid IgG antibody. The dashed box indicates the desired protective threshold concentration. (C) Test results of ISF samples spiked with anti-tetanus toxoid IgG, anti-diphtheria toxoid IgG, or anti-*B. pertussis* toxin IgG, and PBS which was used as a blank control.

The flow characteristics of liquid within the patch were first evaluated using an artificial skin model. For this experiment, the bandage tape was removed from the patch to facilitate visualization of fluid flow through the microchannels and LFIA test strip. Within 2 min of applying suction to the patch, liquid was extracted from the micropores (Figure 3A, ii). The extracted liquid flowed into to patch (via the sampling ports) and through the microchannels (Figure 3A, iii). Upon encountering the LFIA test strip, the liquid was wicked through the strip via surface tension (Figure 3A, iv). As the liquid moved across the conjugate pad, AuNP–anti-human IgG conjugates were reconstituted and transported to the test and control lines. By maintaining a constant vacuum pressure, fluid was continuously extracted from the skin, which served to wash away unbound AuNP–anti-human IgG conjugates from the test strip. After ∼11 min, the strip was fully wetted by the liquid and unbound AuNP–anti-human IgG conjugates were completely removed from the strip, resulting in a negligible background signal (Figure 3A, v). Further experimentation was performed to evaluate the extraction of dermal ISF from human skin and its transport through the skin patch (Figure S3). Similar flow characteristics were observed where dermal ISF could be quickly extracted and transported through the LFIA test strip within 18 min. We attribute the slightly longer time required for full wetting of the test strip and removal of unbound AuNP–anti-human IgG conjugates in human skin compared with the artificial skin model to the higher viscosity of ISF.

### Sensitivity and Selectivity of the Tetanus Lateral Flow Assay

We first assessed the analytical sensitivity of the tetanus assay using ISF simulant spiked with varying concentrations of anti-tetanus toxoid IgG. A test line was generated for samples containing anti-tetanus toxoid IgG at concentrations ≥0.08 IU/mL, where the intensity of line was correlated with the antibody concentration (Figure 3B). Several assay parameters, including the AuNP concentration and antibody concentrations, were optimized so that a test line was generated only when the anti-tetanus toxoid IgG concentration in the sample was ≥0.08 IU/mL, which was used as the protective threshold concentration for ISF, indicating immunity against tetanus infection. This threshold concentration was determined by applying the ratio of anti-tetanus toxoid IgG levels in ISF to blood (0.8) (Figure 2) to the established protective threshold concentration of anti-tetanus toxoid IgG in blood (0.1 IU/mL).^40−42^ No test line was generated for samples containing anti-tetanus toxoid IgG at concentrations <0.08 IU/mL. All the samples generated a dark control line, confirming the validity of the tests.

The analytical specificity of the assay was evaluated by testing ISF samples containing anti-tetanus toxoid IgG, anti-*B. pertussis* toxoid IgG or anti-diphtheria toxoid IgG. Vaccination for diphtheria, pertussis and tetanus is commonly administered as a single dose (Tdap);^43^ therefore, antibodies to diphtheria and pertussis toxoids were selected for specificity testing due to their potential to interfere with the tetanus toxoid antigen.^44^ Measurement of a PBS sample was performed and used as a blank control. As shown in Figure 3C, only the sample containing anti-tetanus toxoid IgG generated both test and control lines, indicating a positive test result. In contrast, only the control line was generated for the samples containing irrelevant antibodies, which was identical to the PBS sample, indicating a negative test result. These results demonstrate that this assay is highly specific to anti-tetanus toxoid IgG and exhibits negligible cross-reactivity with potentially interfering antibodies.

### In Situ Detection of Anti-tetanus Toxoid IgG Using the Skin Patch

**Figure 4:**
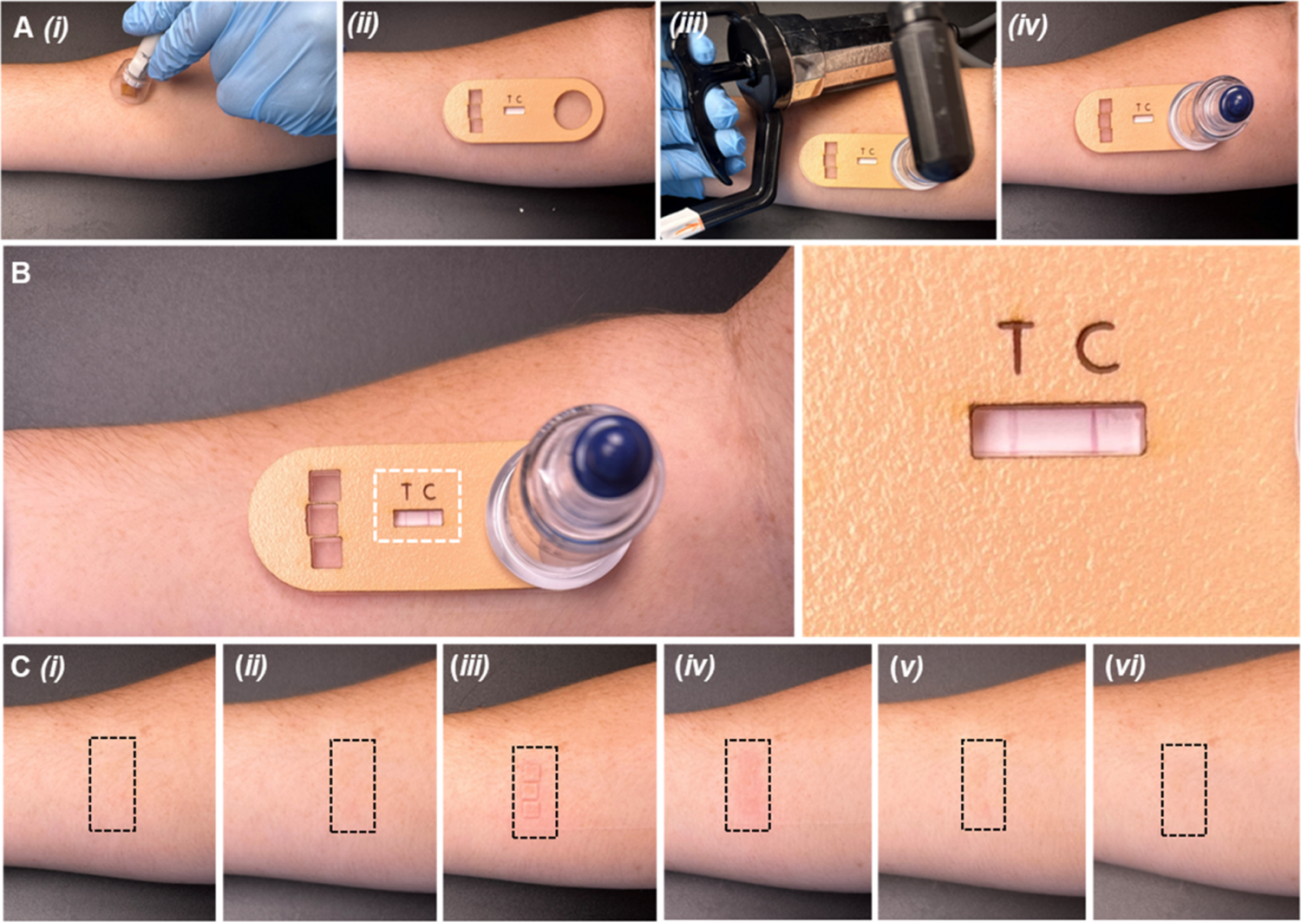
In situ detection of anti-tetanus toxoid IgG in ISF using the skin patch and associated adverse effects. (A) MN insertion in the skin using a MN applicator (i); attachment of the patch to the skin (ii); application of vacuum pressure using a hand pump (iii), followed by removal of the pump and vacuum incubation (iv). (B) Observation of the test results. Inset shows a close-up view of the test result window. (C) Photographs of the forearm of a volunteer before MN insertion (i); immediately following MN insertion (ii); immediately after testing and removal of the skin patch (iii); 5 min after testing (iv); 6 h after testing (v); 24 h after testing (vi). Dashed boxes indicate the MN insertion sites.

To evaluate the functionality of the patch for in situ protein detection, we tested it on a volunteer with an up-to-date tetanus vaccination. To initiate the test, the MN array was first applied to the anterior forearm using a MN applicator (Figure 4A, i). The patch was adhered to the skin in a manner such that the sampling ports were aligned with the MN insertion sites (Figure 4A, ii). Next, a vacuum cup was attached to the vacuum port of the patch and vacuum pressure was generated using a hand pump (Figure 4A, iii). The pump was detached from the cup and vacuum was maintained until ISF was wicked through the entirety of the test strip (Figure 4A, iv). The test results were observable within 18 min of vacuum application (Figure 4B). Both the test line and control line were generated, indicating an anti-tetanus toxoid IgG concentration of ≥0.08 IU/mL, which is consistent with the IgG concentration that was measured in the dermal ISF of this individual (Participant No. 1, Figure 2) using ELISA.

### In Situ Detection of SARS-CoV-2 Neutralizing Antibodies Using the Skin Patch

A second proof of principle demonstration was performed by fabricating another patch designed to detect SARS-CoV-2 neutralizing antibodies and testing it on a volunteer who was recently (within 6 months) vaccinated for SARS-CoV-2. The same testing procedure was used as described above. Both test and control lines were generated within 18 min (Figure S4A), indicating the presence of SARS-CoV-2 neutralizing antibodies in dermal ISF of this individual. For a negative control measurement, this patch was tested on porcine skin dermally injected with ISF simulant. Only the control line was generated on this patch, indicating the absence of SARS-CoV-2 neutralizing antibodies (Figure S4B).

We also investigated whether the use of the patch or testing procedure caused any adverse effects to the skin. MN insertion resulted in slight redness at the MN application sites (Figure 4C, ii). Skin redness and mild swelling localized at the MN insertion sites was observed as a result of suction being applied to the skin (Figure 4C, iii,iv), however, these reactions are common and benign effects associated with vacuum/cupping therapy.^45^ Within 6 h, swelling had completely subsided, and only very faint redness remained (Figure 4C, v). No other reactions were observed, and the redness completed resolved within 24 h after testing (Figure 4C, vi). Overall, testing resulted in very minor adverse effects that were quickly resolved.

## Discussion

Rapid diagnostic testing is used for various applications, including the detection of current or past infections, monitoring disease progression or therapeutic response, and determining immune status to guide vaccination decisions. RDTs enable such testing to be performed outside of laboratory settings by individuals with minimal or no training. Due to their low-cost, quick turnaround time and ease of use, RDTs are widely used throughout the world, particularly in resource-limited settings that lack basic infrastructure and medical resources. However, these tests commonly rely on blood sampling, which poses risks of infection, can lead to complications in infants and individuals with blood disorders, and can deter individuals with blood or needle phobias from getting tested. To address these challenges, this skin patch offers blood-free detection of protein biomarkers in ISF, which can be sampled from the skin in a minimally invasive and nearly painless manner. MN-based biosensing platforms for in situ ISF extraction and analyte detection have previously been reported,^18−24^ however, they required the use of bulky and/or specialized electronic components, such as electrochemical analyzers or custom circuits, and were limited to the detection of small molecules (e.g., metabolites, drugs). In our approach, a MN-based ISF sampling technique is combined with a colloid gold-based LFIA and vacuum-assisted extraction system integrated on a microfluidic skin patch, enabling rapid in situ detection of protein biomarkers in dermal ISF. This device does not require any sample processing (e.g., centrifugation, purification, dilution), resulting in a simplified testing protocol and a reduced risk of disease transmission due to no sample handling. Furthermore, the colorimetric readout enables the test result to be observed by the naked eye without requiring specialized instrumentation. Unlike previously reported ISF sampling techniques that rely on specialized equipment or electric vacuum pumps, this device uses an inexpensive (<$10) vacuum cup and hand pump commonly used in cupping therapy, making it portable and amenable for use in both clinical and point-of-care settings.

A major limitation of existing MN-based ISF sampling techniques is that the collected fluid volumes are too low (1–6 μL)^8,11,46^ for biomolecular analysis using LFIAs, which require at least ∼10 μL of sample for testing. One of the key advantages of this device is its ability to extract larger (14.8 ± 2.9 μL [mean ± SD], Figure S5) amounts of dermal ISF within a short period of time. A comparison of this skin patch with other MN- and vacuum-assisted techniques for sampling ISF from human skin is presented in Table S2. Several strategies were implemented to enhance the ISF sampling efficiency of this device. First, a high-density MN array is used to generate hundreds of micropores in the skin, providing multiple paths for ISF extraction. A major challenge associated with vacuum-assisted ISF sampling is that human skin is highly elastic and easily deforms when vacuum pressure is applied, causing the micropores to close. To overcome this challenge, the microfluidic network is fabricated using a semirigid PMMA substrate, which keeps the skin taut when suction is applied and induces the opening of the micropores, facilitating ISF extraction. We observed that fabricating the microfluidic network from thinner/less-rigid PMMA caused the skin to deform significantly when suction was applied to the patch, resulting in no ISF extraction. Additionally, the adhesive backing of the patch creates an airtight seal with the skin, enabling vacuum pressure to be maintained throughout the test. Combining the use of the vacuum cup with the skin patch to generate suction resulted in the creation of a large pressure gradient across the skin, driving the flow of ISF through the micropores. While applying suction to the skin resulted in minor adverse effects (e.g., slight redness), these effects completely resolved within 24 h. Compared to the adverse reactions and complications that can occur with blood sampling (e.g., pain, bruising, hematoma and thromboembolism),^47,48^ this test is significantly less invasive and safer, which will make it more readily accepted by individuals with blood or needle phobias.

Measurements of IgG antibodies have clinical relevance in determining an individual’s immunity to specific pathogens and guiding vaccination decisions. The ability to determine an individual’s immunity to diseases in a rapid and minimally invasive manner is particularly valuable for individuals who are under-vaccinated or unaware of their vaccination status, putting them at an elevated risk of infection.^49^ In this work, anti-tetanus toxoid IgG and SARS-CoV-2 neutralizing antibodies were used as target biomarkers to demonstrate proof of principle of this technology. Using a MN- and vacuum-assisted sampling technique, ISF was successfully collected from human volunteers. ELISA measurements of ISF and blood samples revealed the presence of anti-tetanus toxoid antibodies in both fluids, where concentrations in ISF were correlated with those in blood. This result is significant since it validates the diagnostic utility of dermal ISF for the detection of disease-specific immune antibodies and protein biomarkers. The functionality of the skin patch was further demonstrated through rapid ISF extraction from human skin and generation of test results in <20 min, showcasing its potential for rapid diagnostic testing.

The parylene-coated SU-8 MNs used in this work offer several advantages, including ease of fabrication, high mechanical strength and excellent biocompatibility. However, MN arrays fabricated from other types of biocompatible materials (e.g., polymers, metals, and ceramics) could also be used for generating micropores in skin. We envision that this device can be readily modified to determine immunity to other diseases, such as diphtheria and pertussis, by replacing the tetanus toxoid protein with a different capture antigen and modifying the assay parameters to adjust the protective threshold concentration. Alternatively, modifications can be made to the assay enabling the detection of other protein biomarkers associated with viral, parasitic, and bacterial infections, such as HIV infection, malaria, Dengue fever or Lyme disease, further expanding the utility of this device for diagnostic testing.

## Supporting Information Available

Design of the MN array and characterization of MN penetration in porcine skin; overview of the ISF sampling procedure; characterization of ISF extraction from human skin and fluid transport through the patch; skin patch test results for the detection of SARS-CoV-2 neutralizing antibodies; characterization of ISF volume extracted by the patch (Figures S1–S5). Demographics of volunteers whose samples were collected and analyzed; comparison of MN- and vacuum-assisted ISF sampling techniques (Tables S1, S2) (PDF)

## Abbreviations

RDT: rapid diagnostic test
ISF: interstitial fluid
LFIA: lateral flow immunochromatographic assay
AuNP: gold nanoparticle
MN: microneedle
LCMS: liquid chromatography mass spectrometry
ELISA: enzyme-linked immunosorbent assay
PDMS: polydimethylsiloxane
PET: poly(ethylene terephthalate)
PMMA: poly(methyl methacrylate)
PBS: phosphate-buffered saline
EDC: 1-ethyl-3-(3-(dimethylamino)propyl)carbodiimide
NHS: *N*-hydroxysuccinimide (NHS)
