# Supplementary material for: Lateral Flow-Based Skin Patch for Rapid Detection of Protein Biomarkers in Human Dermal Interstitial Fluid
Source: ACS Sens. 2024 Oct 25;9(11):5792–801. doi: 10.1021/acssensors.4c00956 (PMC11590092; doi:10.1021/acssensors.4c00956)
Supplement: Supplementary file 1 — se4c00956_si_001.pdf [file se4c00956_si_001.pdf]

Supporting Information

Lateral Flow-Based Skin Patch for Rapid Detection of Protein Biomarkers in Human Dermal Interstitial Fluid

Elizabeth C. Wilkirson[†], Danika Li[‡], Peter B. Lillehoj[*,†,‡]

[†]Department of Mechanical Engineering, Rice University, Houston, TX 77005, USA

[‡]Department of Bioengineering, Rice University, Houston, TX 77030, USA

*Corresponding Author: Peter B. Lillehoj; E-mail: lillehoj@rice.edu

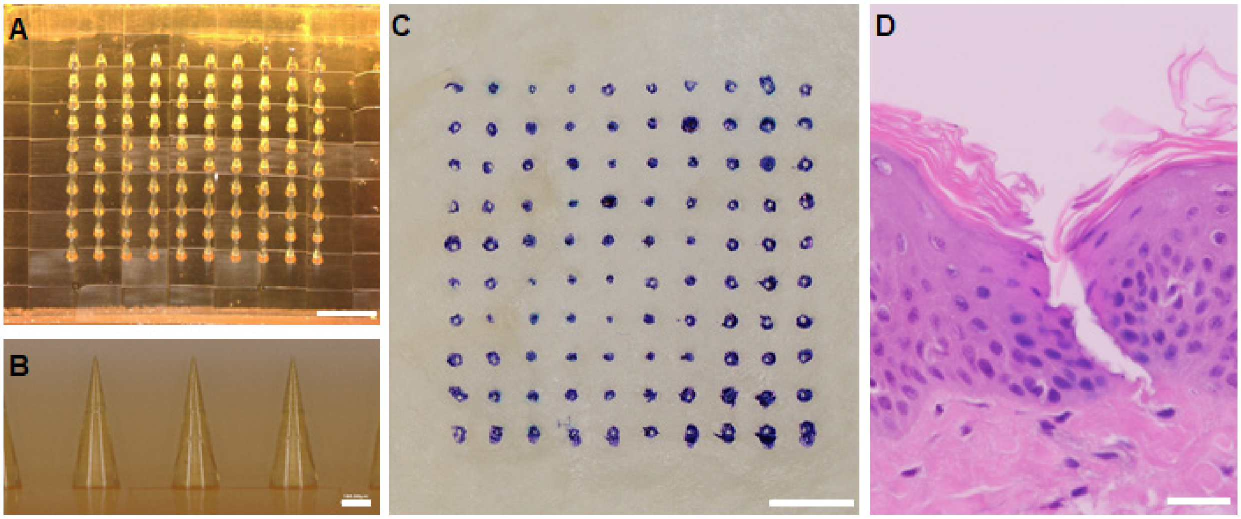

Figure S1. Design of the MN array and characterization of MN penetration in porcine skin. (A) Optical micrograph of the MN array at 20× magnification. Scale bar, 1000 µm. (B) Close-up view of the MNs at 80× magnification. Scale bar, 100 µm. (C) Micropores generated in porcine skin following MN insertion using MNs coated with blue ink. Scale bar, 1000 µm. (D) H&E-stained section of porcine skin penetrated by MNs. Scale bar, 25 µm.

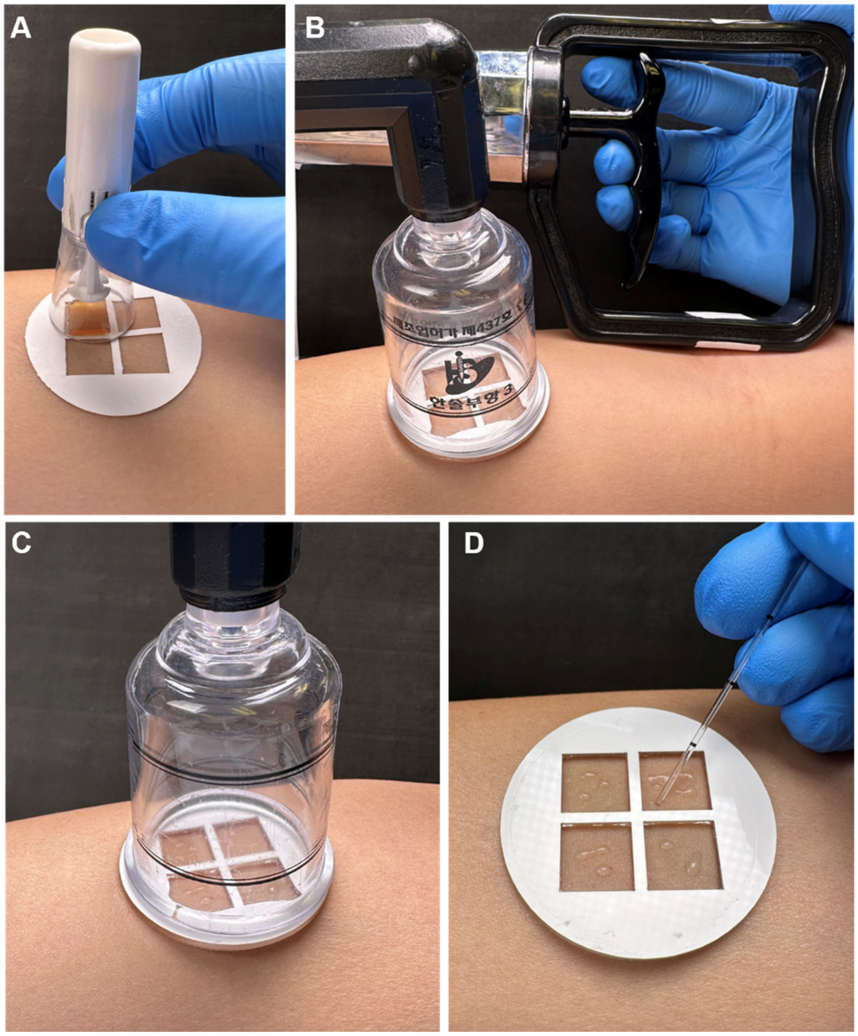

Figure S2. Overview of the ISF sampling procedure on human skin. (A) The stencil is adhered to the anterior forearm, followed by MN insertion using the MN applicator. (B) The PMMA plate is attached to the stencil, followed by the attachment of a vacuum cup. Vacuum pressure is generated in the cup using a hand pump. (C) Vacuum pressure is maintained for 20 min. (D) The vacuum cup is removed and the extracted ISF is collected using capillary tubes.

Table S1. Demographics of volunteers whose blood and ISF were collected and analyzed for anti-tetanus toxoid IgG.

Participant No.	Age	Gender	Ethnicity
1	25	Female	White
2	19	Female	Asian
3	23	Male	White
4	38	Male	Prefer not to answer

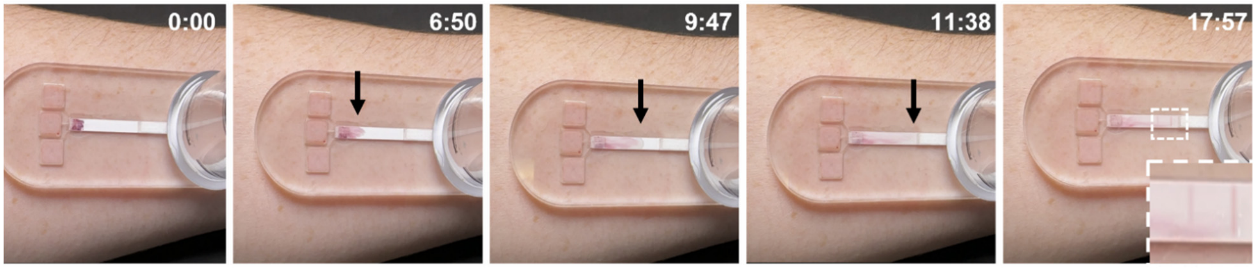

Figure S3. Vacuum-assisted extraction and transport of dermal ISF through the skin patch. Sequential still frame images showing the extraction and transport of ISF through the patch (without the bandage tape) on a volunteer. Arrows indicates the location of the liquid front. Inset shows a close-up view of the test and control lines. Time stamps (min:s) are in the upper right corners.

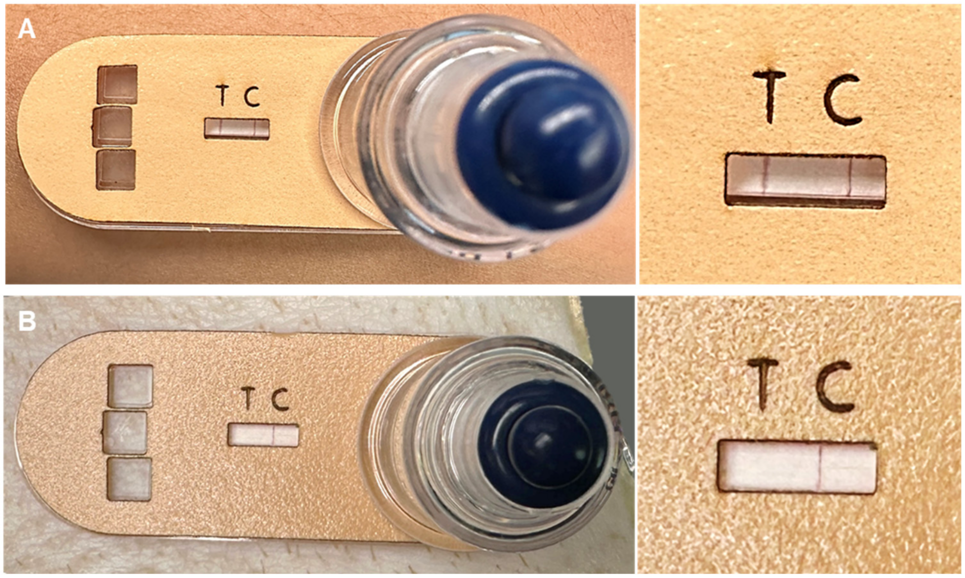

Figure S4. In situ detection of SARS-CoV-2 neutralizing antibodies in ISF using the skin patch. (A) Test result obtained from a volunteer who was recently vaccinated against SARS-CoV-2. (B) Test result obtained from cadaver porcine skin dermally injected with ISF simulant. Insets show a close-up view of the test result window.

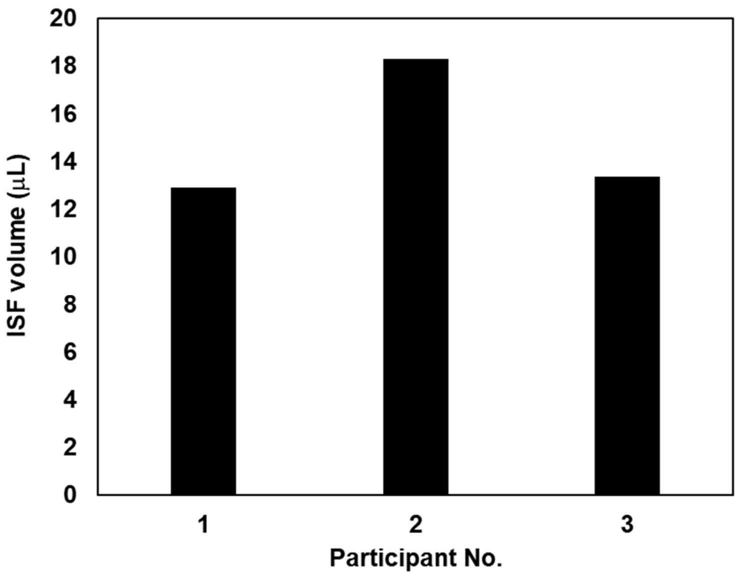

Figure S5. Volume of ISF extracted by the skin patch from three volunteers. Each bar represents a single measurement from a unique participant.

Table S2. Comparison of MN- and vacuum-assisted techniques for sampling ISF from human skin.

Type of MN	Equipment Used for ISF Extraction	ISF Extraction Time	Collection Volume (mean ± SD)	Reference
Solid	Electrical vacuum pump	20 min	2.3 ± 2.1	15
Solid	Electrical vacuum pump	20 min	3.4 ± 3.2	8
Solid	Vacuum cup and hand pump	20 min	20.8 ± 19.4	29
Solid	Vacuum cup and hand pump	18 min	14.8 ± 2.9	This work
